# Proteomics in aging research: A roadmap to clinical, translational research

**DOI:** 10.1111/acel.13325

**Published:** 2021-03-17

**Authors:** Ruin Moaddel, Ceereena Ubaida‐Mohien, Toshiko Tanaka, Alexey Lyashkov, Nathan Basisty, Birgit Schilling, Richard D Semba, Claudio Franceschi, Myriam Gorospe, Luigi Ferrucci

**Affiliations:** ^1^ Biomedical Research Centre National Institute on Aging, NIH Baltimore MD USA; ^2^ Buck Institute for Research on Aging Novato CA USA; ^3^ Wilmer Eye Institute Johns Hopkins University School of Medicine Baltimore MD USA; ^4^ University of Bologna and IRCCS Institute of Neurological Sciences Bologna Italy

**Keywords:** aging, geroscience, human, proteomics

## Abstract

The identification of plasma proteins that systematically change with age and, independent of chronological age, predict accelerated decline of health is an expanding area of research. Circulating proteins are ideal translational “omics” since they are final effectors of physiological pathways and because physicians are accustomed to use information of plasma proteins as biomarkers for diagnosis, prognosis, and tracking the effectiveness of treatments. Recent technological advancements, including mass spectrometry (MS)‐based proteomics, multiplexed proteomic assay using modified aptamers (SOMAscan), and Proximity Extension Assay (PEA, O‐Link), have allowed for the assessment of thousands of proteins in plasma or other biological matrices, which are potentially translatable into new clinical biomarkers and provide new clues about the mechanisms by which aging is associated with health deterioration and functional decline. We carried out a detailed literature search for proteomic studies performed in different matrices (plasma, serum, urine, saliva, tissues) and species using multiple platforms. Herein, we identified 232 proteins that were age‐associated across studies. Enrichment analysis of the 232 age‐associated proteins revealed metabolic pathways previously connected with biological aging both in animal models and in humans, most remarkably insulin‐like growth factor (IGF) signaling, mitogen‐activated protein kinases (MAPK), hypoxia‐inducible factor 1 (HIF1), cytokine signaling, Forkhead Box O (FOXO) metabolic pathways, folate metabolism, advance glycation end products (AGE), and receptor AGE (RAGE) metabolic pathway. Information on these age‐relevant proteins, likely expanded and validated in longitudinal studies and examined in mechanistic studies, will be essential for patient stratification and the development of new treatments aimed at improving health expectancy.

## Graphical abstract

A review of studies that assessed proteins in micro‐specimens of biological fluids or tissues revealed that a substantial group of proteins change systematically with aging. These proteins are strong candidate for developing clinical tools aimed at measuring biological aging and predict changes in health span.

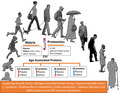

**Abbreviations**
AGE: advance glycation end products
AGRP: agouti related protein
AMPK: AMP‐activated protein kinase
CAT: catalase
CBS: cystathionine‐β‐synthase
CSF: cerebrospinal fluid
DIA: data‐independent acquisition
DiaPASEF: data‐independent acquisitions parallel accumulation‐serial fragmentation
EGFR: epidermal growth factor receptor
FOXO: Forkhead box protein O
GDF15: growth differentiation factor 15
GRB2: growth factor receptor‐bound protein 2
GWAS: genome‐wide association studies
HCys: homocysteine
HIF1: hypoxia‐inducible factor 1
HSC: hematopoietic stem cell
IGF: insulin‐like growth factor
IL: Interleukin
JAK: Janus kinase
LC‐MS: liquid chromatography–mass spectrometry
MAPK: mitogen‐activated protein kinases
MSC: mesenchymal stem/stromal
Mtor: mechanistic target of rapamycin
mTORC1: mechanistic target of rapamycin complex 1
NFkB: nuclear factor κB
PEA: proximity extension assay
PI3 K: Phosphatidylinositol 3 kinase
PTM: post‐translational modification
RAGE: receptor for advanced glycation end products
ROS: reactive oxygen species
SOD2: superoxide dismutase 2
STAT: signal transducer and activator of transcription
TIMP1: metalloproteinase inhibitor 1
TYK2: Non‐receptor tyrosine‐protein kinase
ULK1: UNC‐51‐like autophagy activating kinase 1
VEGF: vascular endothelial growth factor
YWHAQ: 14–3–3 protein theta

## 1 INTRODUCTION

The use of biomarkers to derive multivariable indices of aging and deviation from healthy aging is an expanding area of research that is taking central stage in the geriatric and gerontological literature (Ferrucci et al., 2020). Assessing the aging process by monitoring clinical and biological variables has long‐standing roots in aging research and progress in this field expanded rapidly in recent years. The discovery of the epigenetic clocks by Steve Horvath, Gregory Hannum, and others accelerated a growing interest of researchers in this field (Hannum et al., 2013; Horvath & Raj, 2018). These authors found that the percent methylation of a specific subgroup of CpG sites on DNA extracted from blood or other tissues can predict chronological age with great precision, suggesting that at least part of the aging process is not stochastic and follows a pre‐determined time series of events. Since then, a number of epigenetic clocks have been proposed; the most recent iterations tuned on health‐related variables rather than chronological age appear to predict changes in health and the risk of developing health outcomes more accurately (Levine et al., 2018; Lu et al., 2019). The most recent algorithms reached a degree of predictive accuracy compatible for use in clinical studies (Justice & Kritchevsky, 2020).

**FIGURE 1:**
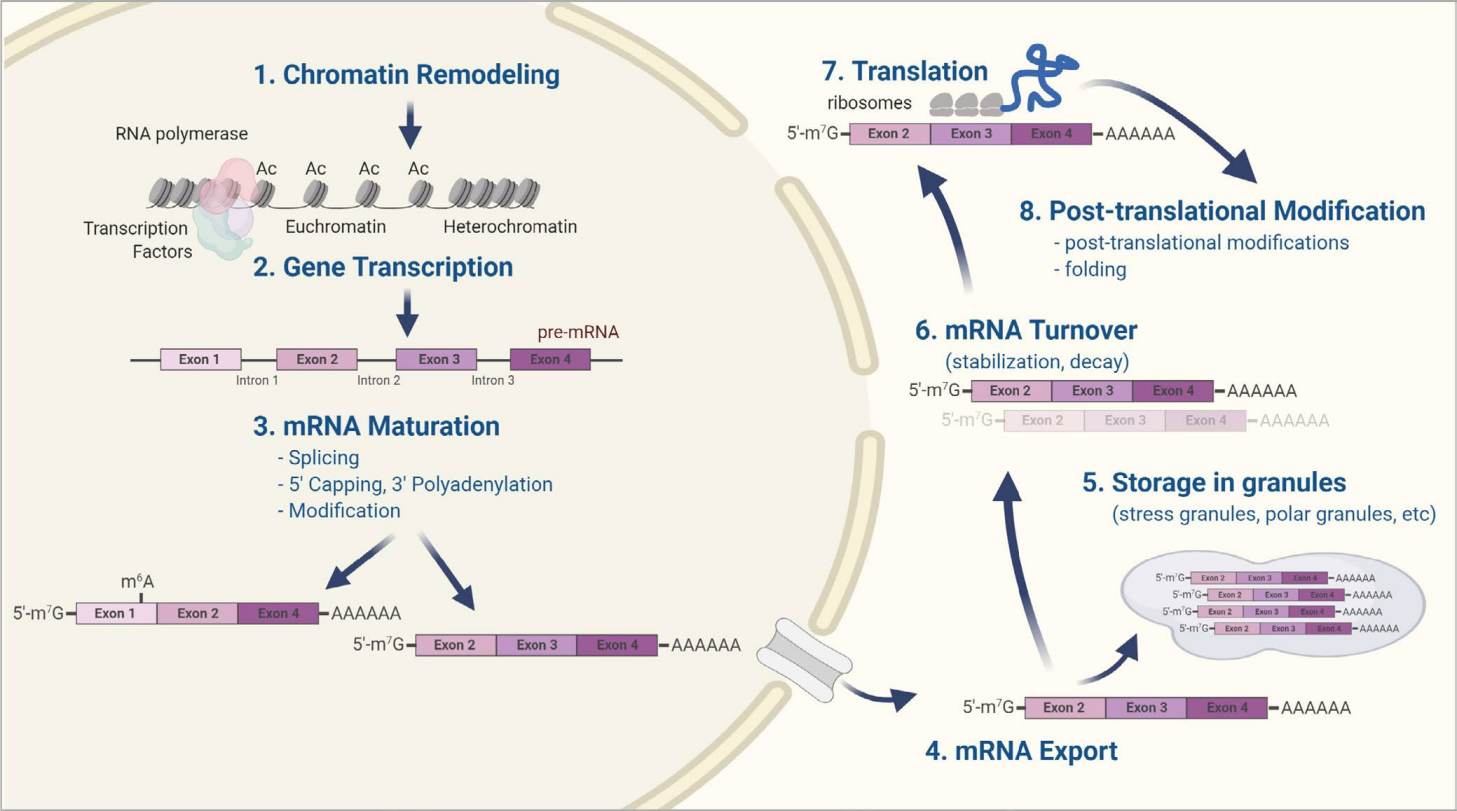
The cascade from transcription to translation is very complex and only a portion of mRNA transcripts are translated into proteins. This explains why measures of transcripts and proteins (and protein functions) are poorly correlated. Several mechanisms regulate gene expression from transcription to translation in order to determine the precise production and timing of a given protein in a cell. (1) DNA wraps around histone proteins in a very tight, non‐accessible structure (heterochromatin). The acetylation of the lysine at the end of histones loosens its organization (euchromatin), eventually allowing transcription factors and RNA polymerase to access DNA and initiate (2) transcription. The RNA polymerase copies a segment of DNA into and antiparallel RNA strand. If the segment of DNA transcribed includes a protein‐coding gene, a pre‐mRNA is produced. (3) Introns are then removed, and exons are assembled into splicing variants; mRNA maturation includes addition of a methyl‐guanosine nucleotide at the 5′ end (“5′ capping”) and addition of multiple adenosines [a poly(A) tail] at the 3′ end, which are necessary for nuclear export, stability, and translation of the mature mRNA. In the cytoplasm, mRNA can be stored in granules (e.g., stress granules or polar granules) and subsequently removed from storage (5) or undergo modifications that substantially reduce or increase turnover (6). Finally, mRNA can be transported to ribosomes and translated into proteins (7). Nascent proteins in turn undergo a number of post‐translational chemical modifications (PTMs), folding and assemblage that strongly affect their biological and properties (8)

**FIGURE 2:**
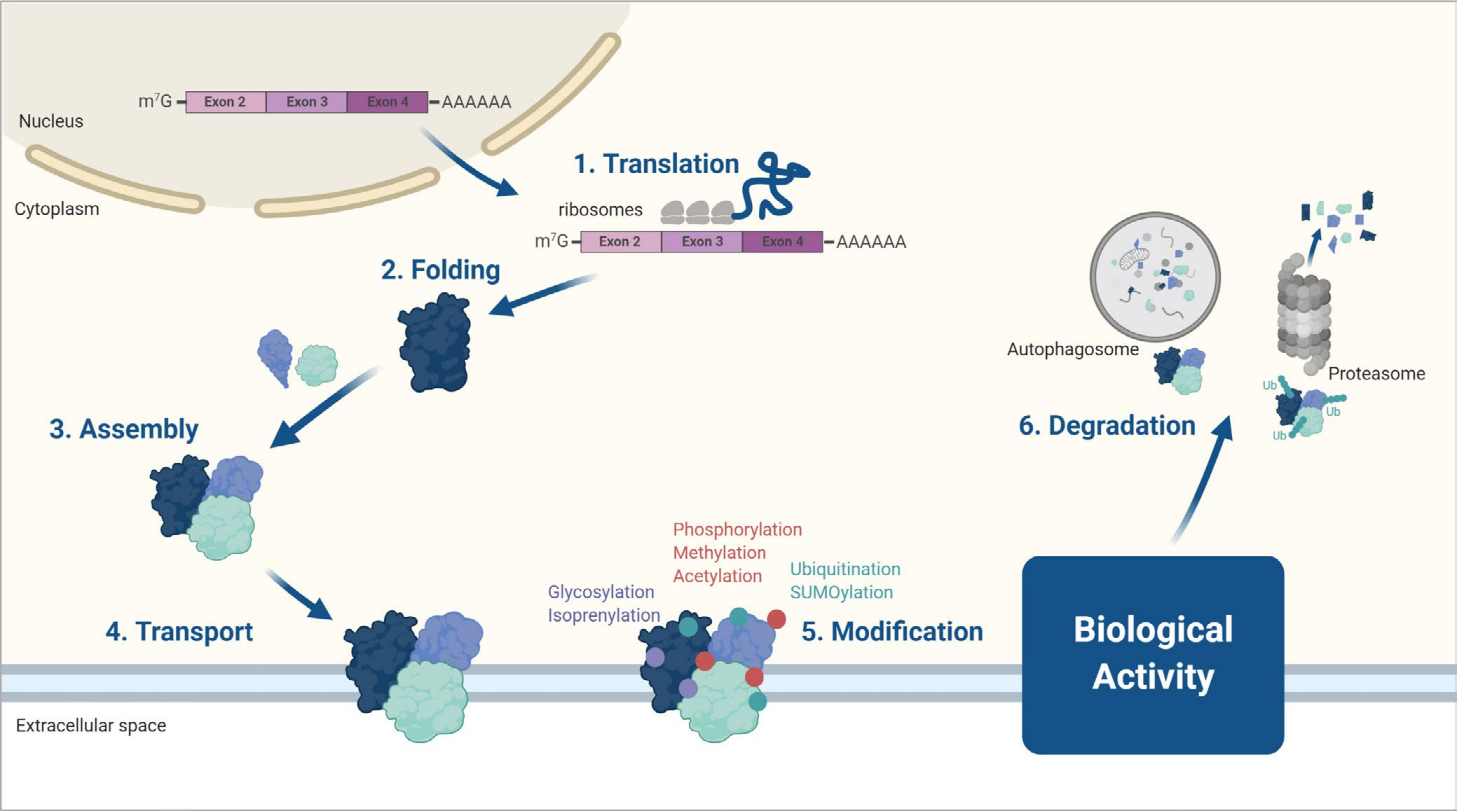
Protein‐level changes are proximal to biological activity biological activity. After translation (1) proteins are folded by chaperones (2), in some cases assembled with other proteins into functional complexes (3), transported to the right site (4), and chemically modified by various additions such as phosphorylation, glycosylation, or acetylation (5). All these modifications can profoundly affect the protein biological activity. Proteins are then recycled after degradation by autophagy and/or the ubiquitin proteasome system (6). Of note, proteins can be quantified at various stages of maturation differently by different methods. For example, aptamers and proximity extension assays require appropriate folding while LC‐MS‐based methods are based on AA sequence

A limitation of epigenetic clocks is the agnostic nature of its strong association with aging and health, which is in part due to our incomplete understanding of the role of DNA methylation in cell biology. The fact that methylation blocks the binding of specific transcription factors is merely one of the many functions that methylation plays in modulating gene expression programs (Dor & Cedar, 2018). Even if we could fully understand the mechanisms by which methylation induces or represses transcription, messenger RNAs undergo a great deal of post‐transcriptional processing that may selectively affect what portion of RNA is translated into proteins (Figure 1). Indeed, attempts to create aging and health “clocks” based on transcriptomic data have been only partially successful and have not reached the precision and predictivity of epigenetic clocks, although research in this field is still evolving (Solovev et al., 2020). Beyond protein translation itself, there are many additional factors that influence the aging proteome, including post‐translational protein changes (see Figure 2) such as post‐translational modifications, protein folding, protein aggregation, protein proteolysis (degradation), protein turnover, protein secretory phenotypes, and which proteins are released or shedded into biofluids (López‐Otín et al., 2013). All of these translation‐independent changes in cells and tissues clearly influence biological activity and processes during aging.

In the thread from genetic information to physiological functions, proteins and metabolites are final effectors responsible for setting phenotypes. Proteins directly affect physiological functions and therefore should offer more informative links to aging and age‐related pathology. Indeed, clinicians use information on circulating levels of proteins and metabolites to diagnose disease in the early preclinical stage, to assess disease severity, to track the effectiveness of medical and surgical interventions, and to make prognostic inferences. Yet, most clinical protocols rely on a single or a handful of protein biomarkers. In addition, while there have been great advancements on the use of proteomic biomarkers in research, the translation to a clinical setting has proved challenging (Ioannidis, 2013). Some of the roadblocks include assays that do not meet the clinical precision requirements and/or the lack of large‐scale evidence that changes in these biomarkers result in important health outcomes. A noteworthy illustration is interleukin‐6 (IL6), the “cytokine for gerontologists” (Ershler, 1993). There is consensus, supported by thousands of published studies, that inflammaging, or the increased levels of pro‐inflammatory markers with aging in blood and tissues, is one of the main drivers of the age‐related increased susceptibility to chronic diseases, multi‐morbidity, and decline of physical function (Ferrucci & Fabbri, 2018). IL6 is by far the best biomarker for measuring inflammaging. Yet, measuring IL6 is still considered a research tool rather than a clinically relevant measure, except for specific diseases (Heikkila et al., 2008).

New proteomic platforms allow the assessment of thousands of proteins from multiple biological matrices, including but not limited to plasma, cerebrospinal fluid (CSF), saliva, skeletal muscle, liver, etc. With few exceptions, the measurement of a single protein will not suffice as a diagnostic or prognostic biomarker for disease diagnosis, with even less ability to capture the complex mechanisms underneath biological aging as well as the complex pathogenesis underneath many age‐related chronic conditions. More recently, a few multiplex protein panels have been approved for use in clinical settings for the risk stratification in lung cancer and preterm birth (Kearney et al., 2018). This supports the idea that a set of proteins, rather than a single biomarker, may have better clinical utility. The elucidation of patterns in changes in protein expression could provide insight into the major mechanisms underlying disease or aging that could be further tested in mechanistic experiments, longitudinal studies, and clinical trials (Johnson et al., 2020). The integration of genetic, proteomic, and metabolomic data in a system‐based analysis of cellular processes could offer significant improvements in predictive medicine.

To this end, a concerted effort has been undertaken to characterize the proteomic signature of chronological and biological age in plasma (Lehallier et al., 2019; Tanaka et al., 2018, 2020). For example, we have recently published a plasma proteomic signature of age that includes 76 proteins highly correlated with chronological age, with growth differentiation factor 15 (GDF15) having the strongest, positive association with age (Tanaka et al., 2018). Several other studies have identified subset of proteins in human tissues and biological fluids that appear to systematically change with aging. A recent review by Johnson and colleagues summarized the findings of 32 of these studies involving over 11,000 participants (Johnson et al., 2020). Despite a large heterogeneity of study populations, tissues examined, and proteomic platforms, these authors identified 1128 proteins that differed systematically by age groups in two or more studies, and only 32 proteins that were replicated in at least five independent studies (Johnson et al., 2020). The identification of a subset of protein concentrations in different tissues may provide new clues for the biological processes and mechanisms underlying the aging process, as well as the mechanisms that connect aging with chronic diseases. In addition, these proteins can potentially be used as longitudinal biomarkers to monitor changes over time in individuals. This new knowledge has the potential to provide the foundations for the development of new, more effective intervention strategies aimed at increasing healthspan. However, a limitation of the Johnson and colleague review was that the direction of protein and age association within each matrix was not considered. An increase in protein expression in plasma, for example, does not necessarily result in a corresponding increase in a different tissue or matrix (Emilsson et al., 2019).

## 2 METHODS

### Analysis of aging proteomes in human plasma and other matrices

Herein, we performed a detailed literature search for proteomic studies in multiple matrices (plasma, serum, urine, saliva, tissues) and species related to aging (Google and PubMed search with the criteria “aging”/”ageing”, “proteomics”, “plasma proteome”/ “aging proteome”, “healthy control”). The manuscripts were screened by four investigators (RM, CU, TT, AL), and each manuscript was reviewed by two investigators. Inclusion criteria for a manuscript included healthy individuals, proteomics results published on or after 2010, publications/authors provided list of significant/all proteins. Thirty three manuscripts met the inclusion criteria, including 12 that covered human plasma (Enroth et al., 2015; Lehallier et al., 2019; Lind et al., 2019; Menni et al., 2015; Ngo et al., 2016; Orwoll et al., 2018; Santos‐Lozano et al., 2020; Sun et al., 2018; Tanaka et al., 2018; Wang, Zhu, et al., 2019; Wang, Zhang, et al., 2019; Ye et al., 2019), 9 from 14 different matrices in humans (Baird et al., 2012; Bakun et al., 2014; Bell‐Temin et al., 2019; Gueugneau et al., 2014; Heinze et al., 2018; Hennrich et al., 2018; Ubaida‐Mohien et al., 2019; Waldera‐Lupa et al., 2014; Wang, Wang, et al., 2018), and 12 publications covering 21 different species/matrices (Amin et al., 2020; Angelidis et al., 2019; Braun et al., 2016; Cutler et al., 2017; Drulis‐Fajdasz et al., 2018; Gebert et al., 2020; Heinze et al., 2018; Kelley et al., 2018; Meng et al., 2020; Stauch et al., 2015; Wang, Zhu, et al., 2019; Yu et al., 2020). The age range of participants included in these studies spanned from 14 to 103 years, and measures were carried out using multiple platforms including but not limited to liquid chromatography–mass spectrometry (LC‐MS)‐based proteomics, multiplexed proteomic assay using modified aptamers (SOMAscan) and Proximity Extension Assay (PEA, O‐Link) (Gold et al., 2010; Lundberg et al., 2011). A total of 4,077 proteins were identified across all human plasma studies.

## 3 RESULTS AND DISCUSSION

We identified 232 proteins whose plasma concentration was significantly associated with age in a consistent direction in at least two different studies and associated with age in at least one other non‐plasma matrix regardless of the direction. The results of the proteins meeting these criteria are presented in Table S1 and are classified into 4 categories: proteins that increased expression in plasma and other matrices with age (125 proteins), proteins that decreased in plasma and other matrices with age (29 proteins), proteins that increased in plasma but decreased in other matrices with age (42 proteins), and proteins that decreased in plasma but increased in other matrices with age (36 proteins) (Table S1). Thus, 154 proteins significantly changed in the same direction in two or more studies in plasma and at least one study in another human matrix. Interestingly, of these 154 proteins, 43 were identified as significantly associated with age in non‐human mammals (Table S1).

### 3.1 Age‐associated signaling pathways

**FIGURE 3:**
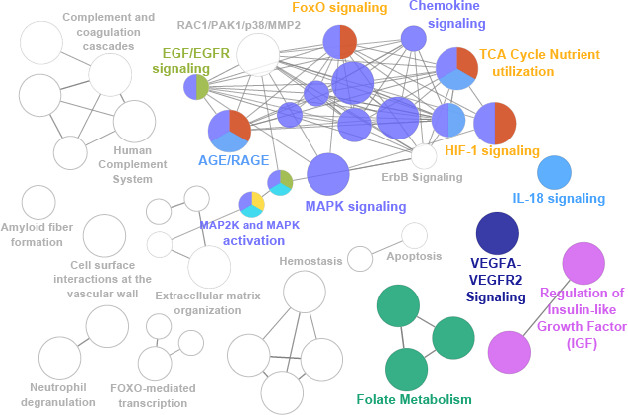
Pathway enrichment analysis of aging proteome. Visualization of 21 pathway clusters from 232 proteins that change systematically with aging (present in 12 human plasma proteome studies and other matrices). Each node (circle) is a pathway, and similar functional groups are clustered together. The representative (most significant) pathways from the pathway clusters are labeled. Pathways discussed here are shown in highlighted colors (IGF, FOXO, AGE/RAGE, MAPKinase, Chemokine/Cytokine, Folate metabolism), and all other pathway groups are shown as gray outlined. All pathways are Bonferroni‐corrected *p* < 0. 05. Node size shows pathway term significance; bigger nodes are most significant. Node color shows the proportions of genes from each cluster that are associated with the pathway

We postulated that the 232 age‐associated proteins found in this study are expressions of the molecular mechanisms underlying aging. To start identifying these molecular mechanisms, we performed an enrichment analysis by ClueGO (Bindea et al., 2009) using the Reactome, Wiki Pathways, and KEGG databases. We limited the search to pathways that included at least 4 genes from the list of the identified age‐associated proteins in this study. Using the 232 age‐associated proteins, 21 pathway groups (112 pathways) were identified (*p* < 0. 05 after Bonferroni correction) (Table S2). Concordant with our hypothesis, the pathways identified describe biological and physiological mechanisms that are widely recognized as important for aging, including several signaling pathways, such as IGF, MAPK, HIF‐1, cytokine signaling pathways and metabolic pathways, FOXO, AGE, and RAGE and folate metabolism (Figure 3). Hereafter, we briefly describe several key significant pathways identified and provide some specific descriptions of the proteins involved and their connections. Of note, we only provide a general description of these pathways, which is in no way exhaustive of their complexity but is mostly focused to underline the many interactions between them. As in any enrichment analysis, the quality of the results is proportional to the quality of available databases, which is often biased and insufficient especially for the identification of pathways and mechanisms involved in aging biology.

#### 3.1.1 Regulation of IGF

The IGF1 signaling is an evolutionarily conserved pathway (Barbieri et al., 2003) that plays a central role in cell growth, survival, maturation, and proliferation and has been shown to be involved in aging and longevity in humans (Barbieri et al., 2003; Wrigley et al., 2017). IGF1 binds to the IGF1 receptor, activating the phosphatidylinositol 3 kinase (PI3 K)‐Akt signaling pathway and promoting cell growth and maturation (Kelley et al., 2018; Stauch et al., 2015). Akt subsequently activates mechanistic target of rapamycin (mTOR), a key kinase in a pathway that significantly correlates with age (Table S2) (Kelley et al., 2018; Stauch et al., 2015).

Interestingly, in humans IGF1 declines with aging and higher levels are associated with higher muscle strength and lower mortality. However, in other species, downregulation of IGF1 signaling has been shown to increase lifespan (Wrigley et al., 2017). In this review, IGF1 signaling was found to decrease with age in both human plasma and CSF (Table S1), while it did not significantly change in other species. IGF1R activates Ras and triggers the MAP kinase pathway through the Sch protein. MAPKs play a role in many cellular processes including gene expression, mitosis, motility, apoptosis, differentiation, and metabolism (Cargnello & Roux, 2011; Manna & Stocco, 2011). In our literature review, we found that proteins representing MAPK signaling pathway significantly correlated with age, consistent with studies demonstrating that aging strongly enhances MAPK activities (Kim et al., 2002). In particular, MAPK1 and MAPK3 were overrepresented with increased age in human plasma, human skeletal muscle, mouse hippocampi, and white adipose tissue (Table S1).

#### 3.1.2 FOXO signaling pathway

FOXO proteins are a subfamily of the Forkhead family of transcription factors that are involved in many cell processes, including cell metabolism, growth, differentiation, apoptosis, oxidative stress, senescence, autophagy, stress resistance, and energetic metabolism (Lee & Dong, 2017; Martins et al., 2016; Murtaza et al., 2017). Insulin or IGF‐1 triggers the phosphorylation of FOXO factors by AKT, leading to increased FoxO binding to the regulator protein 14–3–3 and the consequent export of FoxOs from the nucleus and suppression of FOXO‐dependent transcription (Martins et al., 2016; Morris et al., 2015). There is robust evidence that FOXO pathway is important for aging (Akasaki et al., 2014). In *C*.*elegans*, a null mutation of the FOXO ortholog DAF‐16 is associated with life extension, likely through the modulation of carbohydrate metabolism and by enhancing stress response (Murtaza et al., 2017; Ogg et al., 1997). In humans, genetic variations of FOXO3A are associated with healthspan and longevity and are highly prevalent in centenarians (Schork & Raghavachari, 2018). Interestingly, in this literature review, we found several age‐related proteins regulated by FOXO transcription factors (Morris et al., 2015), including proteins implicated in energy homeostasis (agouti‐related neuropeptide (AGRP)), ROS detoxification (catalase (CAT), superoxide dismutase 2 (SOD2)), and epidermal growth factor receptor (EGFR) signaling pathway (Table S2).

#### 3.1.3 AGE and RAGE

AGEs are proteins or lipids that become glycated when exposed to high concentrations of sugars. Small amounts of AGEs are formed under physiologic conditions, and the concentration of AGEs increases significantly with older age. Higher AGE concentration is observed in diabetic patients and is associated with increased risk of cardiovascular diseases and premature death (Semba et al., 2009, 2010; Yang et al., 2013; Zhao et al., 2014). The binding of AGEs to their specific receptors (RAGEs) activates several signaling pathways that we found in the enrichment analysis (Table S2). For example, RAGE‐mediated signaling has been shown to be necessary for downstream activation of HIF1 (Khan et al., 2018) and has been shown to activate MAPK, NAD(P)H oxidase and SRC resulting in the phosphorylation and activation of the IGF1 receptor, leading to the activation of Akt (Yang et al., 2013). It was also shown that IGF1 receptor, PI3K, and Akt may be involved in AGEs effects on adipogenesis (Yang et al., 2013).

#### 3.1.4 HIF1 signaling pathway

HIF1, a heterodimeric transcription factor including alpha and beta subunits, is the master intracellular oxygen sensor. Hypoxia blocks the ubiquitination and proteasome degradation of HIF1, which is shuttled to the nucleus and induces the transcription of more than 100 genes involved in adaptive processes that increase oxygen supply and support anaerobic ATP generation, such as angiogenesis, erythropoiesis, anaerobic glycolytic metabolism, pH regulation, cell proliferation, cell survival, inflammation, and immunity (Yeo, 2019). There is extensive evidence that dysregulation of HIF1 occurs with aging and is involved in the pathogenesis of many age‐related chronic conditions, including cancer growth and metastasis, cardiac disease, pulmonary disease, liver disease, and kidney disease (Yeo, 2019). Interestingly, HIF1α downregulates mitochondrial biogenesis, thereby reducing ATP production, and possibly further contributing to the oxidative stress that triggers the nuclear factor κB (NFkB)‐mediated production of cytokines (Yeo, 2019). It is noteworthy that several biological pathways identified by age‐associated circulating proteins are connected with the HIF1 signaling pathway, including vascular endothelial growth factor (VEGF), MAPK1, and STAT3, which increased in plasma and in human mesenchymal stem/stromal (MSC), skeletal muscles, and hematopoietic stem cell (HSC), respectively; MAPK1 and signal transducer and activator of transcription 3 (STAT3) were also found to increase in mouse hippocampi and white adipose tissue, respectively. Hexokinase 1 decreased with age in human plasma, while it increased in human MSC, HSC, and rhesus macaques hippocampus (Table S1) (Beek et al., 2019). Similarly, several signaling pathways triggered by pro‐inflammatory cytokines and chemokines identified in the enrichment analysis may be directly or indirectly affected by HIF1 activity (Tables S1 and S2). Finally, the HIF1 pathway cross‐talks with other pathways that are relevant for aging, including sirtuins, AMP‐activated protein kinase (AMPK), mechanistic target of rapamycin complex 1 (mTORC1), UNC‐51‐like kinase 1 (ULK1), NFκB pathways, suggesting that chronic or episodic hypoxia may be connected to biological aging (Beek et al., 2019; Yeo, 2019).

#### 3.1.5 Folate metabolism

Enrichment analysis showed that there were 15 proteins related to folate metabolism among the age‐associated proteins. Folate along with other B‐vitamins and nutrients are processed through the one‐carbon metabolism, which plays a critical role in maintaining normal metabolic, energy, differentiation and growth status of all mammalian cells (Morris et al., 2015). The one‐carbon metabolism pathway is involved in several biochemical processes including purine and pyrimidine production, mitochondrial protein translation, homocysteine (HCys) remethylation cycle, and transsulfuration pathway (Akasaki et al., 2014). Cystathione‐β‐synthase (CBS) the first enzyme in the hepatic transsulfuration pathway catalyzes Hcy to cystathionine via cysteine and is predominantly expressed in the liver, and its expression has been shown to decrease gradually with cellular aging leading to endothelial dysfunction (Hayden & Tyagi, 2004; Price et al., 2018). In this literature review, a decrease in circulating levels of cystathionine‐β‐synthase (CBS) was observed in plasma with age (Zhao et al., 2014). Decreasing CBS activity/levels could decrease circulating levels of glutathione and result in oxidative stress (Price et al., 2018). Consistent with oxidative stress, in this study, significant correlations were also observed for other endogenous antioxidant enzyme family members, mitochondrial superoxide dismutase, SOD2, and CAT (Hayden & Tyagi, 2004), where they significantly decreased with age in plasma and liver. An overlap between proteins implicated in folate metabolism and proteins in immune related pathways was observed, consistent with recent studies (Ogg et al., 1997; Schork & Raghavachari, 2018).

#### 3.1.6 Cytokine signaling pathway

Cytokines and chemokines are elements of a complex network of the signal transduction machinery that regulates both innate and adaptive immune functions, including cell recruitment and growth (Fulop et al., 2006; Turner et al., 2014). The imbalance of cytokines between the pro‐inflammatory and anti‐inflammatory control mechanisms or inflammaging is a characteristic of aging and aging‐related diseases (Franceschi et al., 2000; Rea et al., 2018). The enrichment analysis performed in this literature review indicated that pathways that involve pro‐inflammatory cytokines (IL‐12, IL‐1, IL‐17, IL‐6 and IL‐18), anti‐inflammatory cytokines (IL‐10 and IL‐13), chemokines, and growth factors (VEGF, IL‐2 and IL‐5) are dysregulated with aging. The profound changes in the cytokine milieu with aging explains, at least in part, the activation of JAK/STAT, Ras‐MAPK signal cascades as well as the activation of the PI3 K‐Akt pathway that promotes cell survival, proliferation and metabolism (Fulop et al., 2006; Slack, 2017). Many proteins involved in the cytokine–chemokine signaling such as CRKL, growth factor receptor‐bound protein 2 (GRB2), MAPK1, MAPK3, STAT1, STAT3, metalloproteinase inhibitor 1 (TIMP1), and 14–3–3 protein theta (YWHAQ) were increased with older age in plasma and other human matrices while non‐receptor tyrosine‐protein kinase TYK2 (TYK2) decreased with older age in plasma, while increasing in HSC (Hennrich et al., 2018).

### 3.2 Age‐related changes in proteoforms (PTMs and protein variants)

Between protein variants and post‐translational modifications (PTMs), a single protein may have hundreds or even thousands of variations called proteoforms (Smith et al., 2013), with different and even opposite biological functions, with very different effects on aging and longevity (Turner et al., 2014). From 20,300 human genes, there are upwards of hundreds of thousands of distinct proteoforms arising from splice variants and PTMs (Aebersold et al., 2018). Therefore, proteoforms represent an entirely new layer of complexity to proteomic analysis and remain an area of aging proteomics with great opportunity for exploration, both in terms of biology and biomarkers.

PTMs play a fundamental role in regulating a broad range of cellular processes including signaling, gene expression, protein localization, and turnover, among many others. Numerous studies have implicated PTMs in the biology of aging and aging‐related diseases including metabolic syndrome, neurodegeneration, and heart disease. Alterations in PTM levels are strongly correlated with age in multiple animal and disease models (Baldensperger et al., 2020; Meyer et al., 2018; Santos & Lindner, 2017) and linked to age‐related diseases in human studies (Chaudhuri et al., 2018; Mnatsakanyan et al., 2018). For example, accumulation of AGEs and oxidative modifications are hallmarks of aging and metabolic diseases (Chaudhuri et al., 2018; Reeg & Grune, 2015). Notably, glycated hemoglobin (HbA1c) is an example of a widely used PTM biomarker for diabetes (Selvin et al., 2010). HbA1c is increased in diabetes due to prolonged exposure to elevated blood glucose, and unlike fasting glucose levels, provides information on the average level of blood sugar over the past 2 to 3 months (Selvin et al., 2010). Even after adjusting to baseline fasting glucose levels, HbA1c is associated with risks of cardiovascular disease and all‐cause death, suggesting a biomarker potential in several chronic diseases of aging (Selvin et al., 2010). Outside of HbA1c, there are a variety of potential protein targets with many potential AGE modifications. Our meta‐analysis of plasma aging biomarkers identified the AGE/RAGE pathway (Figure 3, Table S2), and AGEs may therefore be a promising PTM to examine in future aging biomarker studies in human plasma. Likewise, distinct changes in several types of acylation modifications are altered in aging and metabolic syndrome (Baldensperger et al., 2020; Carrico et al., 2018; Meyer et al., 2018). Yet, little is known about the role these and other modifications play in the progression of aging and related diseases. Granular knowledge about protein targets and modification sites, rather than bulk PTM levels, is lacking. Understanding how specific post‐translationally modified proteins and sites change in aging and diseases is critically needed in order to gain mechanistic understanding of the role of PTMs in aging and identify potential therapeutic targets for translation to humans.

Given the complexity of aging and age‐associated diseases, it is unlikely that single proteins, molecules, or single‐omic panels will be sufficient as diagnostic or treatment biomarkers. The use of multi‐omic signatures that include PTMs may aid in identifying disease‐specific molecular signatures with greater precision (Mnatsakanyan et al., 2018). For example, in the cancer field, EGFR tyrosine kinase inhibitors (TKIs) are used for the initial treatment of lung cancer, for which the primary biomarker is EGFR TKI‐sensitizing mutations. However, 30%–40% of patients have primary resistance to treatment with TKIs (Maemondo et al., 2010; Rosell et al., 2012; Wu et al., 2014; Zhou et al., 2011), underscoring a need for additional biomarkers of treatment response. Phosphoproteomic studies in EGFR‐derived human lung adenocarcinoma cell lines have identified multiple phosphosite indicators of TKI sensitivity, including phosphorylation of kinases (EGFR‐Y1197 and MAPK7‐Y221) and multiple adaptor proteins (Zhang et al., 2017). Collectively, these results suggest the need for studies that examine the value of integrating genomics and phosho‐proteomics as biomarkers of TKI response in human lung cancer. Similarly, the Clinical Proteomic Tumor Analysis Consortium (CPTAC) has integrated large‐scale proteomics and phospho‐proteomics to identify distinct profiles in 77 genomically annotated breast cancer tumors (Mertins et al., 2016). In that study, multiple genes that drove proteogenomic changes similar to HER2 were identified, including CDK12, TLK2, PAK1, and RIPK2, which could potentially represent druggable kinases beyond HER2. However, further studies will be needed to determine the value of these findings in identifying treatments and predicting treatment response.

While proteins are gaining acceptance as clinical biomarkers, the use of PTM biomarkers is in its infancy. Aside from a few prominent examples, such as glycated hemoglobin in diabetes (Selvin et al., 2010), PTMs are rarely used as clinical biomarkers, and PTM signatures are never used clinically. The measurement of proteins alone without regard to isoforms or modifications may not be sufficient or may even be misleading in terms of biomarker discovery. The use or addition of PTM information to proteomic biomarkers may be important for the development of precision biomarkers (Mnatsakanyan et al., 2018). More and more interest in potential PTM biomarkers in disease and aging have been discussed, and clinical mass spectrometry workflows are explored to discover PTM signatures in clinical samples (Mnatsakanyan et al., 2018). Given that many candidate PTMs have been identified for aging and age‐related diseases, discovering and validating PTM biomarker candidates in human systems and clinical samples, either alone or in combination with other proteomic biomarkers, is a promising direction of research going forward. Specific interest for serum and plasma markers may be directed toward N‐glycosylation profiling as markers of physiological age (Vanhooren et al., 2015).

The discovery and validation of PTM biomarkers lends itself nicely to unbiased MS‐based proteomic approaches. Global chemical protein modifications yielding increased protein damage have been a long‐standing hypothesis for aging and could potentially be used as markers. With recent developments in PTM‐analysis pipelines, large‐scale discovery and quantification can be performed on multiple PTMs within a single sample, thus making it feasible to perform experiments that were largely limited by the quantity of protein in a sample, time, and cost (Basisty et al., 2018; Mertins et al., 2013). Secondly, the emergence of new MS acquisition strategies, for example, data‐independent acquisition (DIA) methods can be routinely used for unbiased and quantitative proteomics (Collins et al., 2017). Numerous software pipelines now exist for the analysis of DIA data, and quantitative analysis of PTMs on the site level and workflows for site‐localization are available (An et al., 2019; Xie et al., 2020). Finally, DIA nicely lends itself to discovery proteomics, particularly with PTMs, because once collected, DIA data can be re‐analyzed and re‐searched for new proteins and protein modifications, assuming the corresponding spectral libraries are available. Pipelines for the discovery of protein isoforms resulting from splice variants have also emerged over the last decades with a particular interest in cancer biomarker research (Komor et al., 2017). Protein variants as biomarkers likely will become a highly active research field to bring forward aging biomarkers. One of the difficulties of proteomic analysis of protein variants is a lack of tryptic peptides spanning exon–exon junctions, suggesting a need for complementary digestion methods (Wang, Codreanu, et al., 2018). Proteogenomic pipelines, which integrate RNA sequencing data and MS‐based proteomics to identify differentially expressed protein isoforms, are exciting technological developments that will require exploration in future in biomarker research (Carlyle et al., 2018; Komor et al., 2017; Nesvizhskii, 2014).

### 3.3 Highlights and future directions

Here we presented a list of 232 proteins with strong evidence of age association (Table S1) and describe the pathways these proteins represent (Table S2). Many of the plasma proteins that were found consistently and significantly changing with aging in the literature are enriched for biological pathways known to be important for aging both in animal models and in humans. However, the biological role of some of these specific proteins and their role in the context of aging remain to be fully elucidated in mechanistic studies, longitudinal studies, and clinical trials. The quest to identify the role of these proteins is expected to uncover mechanisms of aging likely important for human health. Moreover, it is also crucial to investigate which of these proteins are most robust, reproducible and show highest selectivity and sensitivity in the applied or projected analytical assays. Of note, these proteins are merely a subset of important aging proteins, and many more were not measured by proteomic platforms queried in this research. Instead of only identifying proteins linearly correlated with aging, the identification of those that capture deviations from normal aging would be of greater interest, either because of accelerated aging or age‐related chronic diseases, similar to the improvements in the second generation of epigenetic clocks (Levine et al., 2018; Lu et al., 2019). Studying non‐linear age association may be a key to identifying important aging proteins. Previous studies, including several discussed in this review, have already shown that some proteins appear to change with aging following different trajectories, most typically showing homogeneous linear change across the lifespan or a period of relative stability followed by rapid increase after a certain age (Lehallier et al., 2019). Trajectories for each specific protein should be fully characterized and classified in cross‐sectional studies as this information will be critical to modeling longitudinal trajectories across proteins and their association with changes of health dimensions. Specific emphasis should also be paid to develop robust and selected “protein panels” as biomarkers of aging or to indicate aging trajectories—as these combinatorial protein panels may better account for human biological variability and heterogeneity in different cohorts. The identification of over 5000 proteins in a few drops of blood or milligrams of tissue, while impressive, is far from providing exhaustive insight, as every single protein may have hundreds of splicing variants, with different and opposing biological functions, with possible divergent effects on aging and longevity (Bhadra et al., 2020).

Current technologies and analytical methods for the identification of splice variants are still not adequate for discovery studies in large populations, although there is progress in this field (Kasianowicz et al., 1996; Meller et al., 2000). Only a handful of technologies are available for measuring proteins in plasma or serum, the most accessible and routinely used biological material in humans, and the one already used routinely in clinical practice. Currently, most of the work on aging used aptamer‐based arrays (SOMAlogic) or PEA multiplex technology (O‐Link) but LC‐MS‐based technologies are being developed that are expanding both in the number of assessed proteins and reliability of the assay (Rice et al., 2019). While MS‐based approaches are widely applied as a tool for plasma biomarker discovery, they are not as widely applied in aging cohorts. A major hurdle in achieving similarly deep coverage of the plasma proteome in MS‐based analysis is the large dynamic range of protein concentrations, where a handful of highly abundant plasma proteins interfere with the detection of low‐abundant plasma proteins. Recently, this has been mitigated in several ways, including abundant protein depletion, fractionation, and newer acquisition schemes (Geyer et al., 2017). An advantage of discovery MS approaches is the unbiased approach and its ability to identify post‐translationally modified proteins, as novel biomarker candidates.

The use of these multiple platforms for the screening of an initial list of proteins is perfect for discovery studies. This was clearly demonstrated in this review where multiple proteins were identified in more than one platform. However, their use in large‐scale studies to elucidate proteomic signatures before they can be employed in the clinic are hampered by high cost and by issues surrounding intellectual property issues pertaining to proteomic signatures proposed for clinical application. In addition, the availability of large databases and phenotypically well‐characterized heterogeneous populations are needed to make progress in this field. New analytical tools, including deep learning and artificial intelligence, are anticipated to help meet this challenge (Kearney et al., 2018). Regardless of the method used for the discovery of biomarker candidates, once the list of critical proteins has been compiled, they can be measured by less expensive and more traditional methods, such as antibody‐based arrays or targeted LC‐MS/MS parallel reaction monitoring methods. This important switch will require a strong collaboration between scientists that have the tools and the knowledge to provide the initial list and companies that can develop, validate, and standardize less expensive and more precise and quantitative methods that can be introduced in large epidemiological and clinical studies. One advantage of this approach is that it could be used concurrently in tissues and in biological fluids while currently LC‐MS/MS is the method of choice for studying proteins in tissue. This is important because one of the lingering questions about blood biomarkers is whether and to what extent they reflect molecular changes that occur in tissues, and this comparison would benefit enormously from using the same technology.

Apart from their utility as biomarkers, an exciting question in geroscience is to what extent blood proteins and other bioactive molecules are involved in driving aging processes, such as cellular senescence (Basisty et al., 2020a). Studies utilizing heterochronic parabiosis and other blood exchange systems in mice have suggested the existence of pro‐geronic and anti‐geronic factors, circulating factors with either pro‐ or anti‐aging effects (Mehdipour et al., 2020; Smith et al., 2018; Villeda et al., 2014). One of the potential sources of pro‐geronic factors in humans is senescent cells (Basisty, Kale, Jeon, et al., 2020; Basisty, Kale, Patel, et al., 2020; Schafer et al., 2020; Tanaka et al., 2018). Therefore, it will be exciting to see to what extent aging biomarkers in the blood can translated into therapeutic targets, or biomarkers for the development of therapeutics such as senolytic and senomorphic drugs.

## 4 CONCLUSIONS

The search for measures of biological aging has stimulated broad interest in aging research and has expanded opportunities for understanding diseases. In this field, the study of proteins has a strong translational potential, since protein concentrations have been established by clinicians for diagnostic purposes for many years and because proteins tend to be direct effectors, with biological mechanisms that are relatively easier to recognize than other omics biomarkers. A substantial amount of work remains to be done before this line of science is applied clinically on a wide scale. However, the intellectual power, the technology and the will are present and give us great hope for the future.

## CONFLICT OF INTEREST

The authors declare no conflict of interest.

## AUTHORS CONTRIBUTIONS

The initial idea for this review was from L. F. R. M, C. U., T. T., and A. L. did the literature searches and created the list of proteins. R. M., C. U., and L. F. wrote the initial draft, including its organization. All authors had significant input into the manuscript. L. F. supervised and planned the writing of the manuscript.

## Supporting information


**Table S1**

**Table S2**
